# Is ChatGPT a Reliable Source of Patient Information on Asthma?

**DOI:** 10.7759/cureus.64114

**Published:** 2024-07-08

**Authors:** Dalal M Alabdulmohsen, Mesa A Almahmudi, Jehad N Alhashim, Mohammed H Almahdi, Eman F Alkishy, Modhahir J Almossabeh, Saleh A Alkhalifah

**Affiliations:** 1 Internal Medicine, College of Medicine, King Faisal University, Hofuf, SAU; 2 Hematology and Oncology, King Saud Medical City, Riyadh, SAU; 3 Internal Medicine, King Faisal General Hospital, Hofuf, SAU; 4 Endocrinology, King Saud Medical City, Riyadh, SAU; 5 Internal Medicine, Abqaiq General Hospital, Abqaiq, SAU; 6 Internal Medicine, Almoosa Specialist Hospital, Al Mubarraz, SAU

**Keywords:** large language models, medical education, reliability, readability, patient information, chatgpt, asthma, artificial intelligence

## Abstract

Introduction: ChatGPT (OpenAI, San Francisco, CA, USA) is a novel artificial intelligence (AI) application that is used by millions of people, and the numbers are growing by the day. Because it has the potential to be a source of patient information, the study aimed to evaluate the ability of ChatGPT to answer frequently asked questions (FAQs) about asthma with consistent reliability, acceptability, and easy readability.

Methods: We collected 30 FAQs about asthma from the Global Initiative for Asthma website. ChatGPT was asked each question twice, by two different users, to assess for consistency. The responses were evaluated by five board-certified internal medicine physicians for reliability and acceptability. The consistency of responses was determined by the differences in evaluation between the two answers to the same question. The readability of all responses was measured using the Flesch Reading Ease Scale (FRES), the Flesch-Kincaid Grade Level (FKGL), and the Simple Measure of Gobbledygook (SMOG).

Results: Sixty responses were collected for evaluation. Fifty-six (93.33%) of the responses were of good reliability. The average rating of the responses was 3.65 out of 4 total points. 78.3% (n=47) of the responses were found acceptable by the evaluators to be the only answer for an asthmatic patient. Only two (6.67%) of the 30 questions had inconsistent answers. The average readability of all responses was determined to be 33.50±14.37 on the FRES, 12.79±2.89 on the FKGL, and 13.47±2.38 on the SMOG.

Conclusion: Compared to online websites, we found that ChatGPT can be a reliable and acceptable source of information for asthma patients in terms of information quality. However, all responses were of difficult readability, and none followed the recommended readability levels. Therefore, the readability of this AI application requires improvement to be more suitable for patients.

## Introduction

Asthma is a chronic disease affecting children, adults, and even older individuals. The condition is globally prevalent, with about 300 million patients worldwide [1]. Symptoms include coughing, wheezing, and shortness of breath, which can all vary in frequency depending on how controlled the patient is [2]. Most asthmatic patients have mild asthma and can live a normal life with an appropriate management plan. However, even mild cases can exacerbate and become life-threatening [3]. The most common trigger for these severe exacerbations is viral infection, yet exposure to allergens and irritants is also a common cause [4]. Notably, adequate asthma control can reduce the risk of acute exacerbations and the need for admissions [5]. The extent to which an individual can acquire and comprehend health information, known as health literacy, is directly related to better asthma control. This means that the ability to access health resources has an impact on the outcome of patients’ health [6].

Given the significant impact of health literacy on patient outcomes, it is crucial to explore how patients access and comprehend health information. While patients have relied on Internet search engines as the main source for answering inquiries regarding their health conditions, these are currently being challenged by artificial intelligence (AI) large language models (LLMs) as the new dominant source. Among these AI models, OpenAI’s (San Francisco, CA, USA) ChatGPT was the fastest-growing, with over 100 million monthly users shortly after its release [7]. Despite the fact that the underlying technology behind ChatGPT may not be novel, its key differentiator lies in its unrestricted public access, free of charge. Moreover, the user-friendly interface and extensive dataset combine to create a unique user experience for interacting with LLMs [8]. So far, the application has gained users’ satisfaction when it comes to obtaining answers about their disorders [9]. Therefore, it is predicted to become a go-to tool for many.

While the release of ChatGPT offers promising new avenues for information access, it also broadens public exposure to a vast amount of data. This necessitates careful evaluation of the presented material's accuracy, particularly when it concerns medical advice [10]. A very recent study (Soto-Chávez et al., 2024) was conducted to evaluate ChatGPT’s answers to a number of questions concerning different chronic internal medicine illnesses, including diabetes mellitus, heart failure, and chronic kidney disease. The study concluded that the tool was able to provide a reliable source of information for these diseases, although it needs improvements in terms of readability [11]. The presented study aims to assess the same tool, ChatGPT, as a resource for asthmatic patients’ informational needs.

## Materials and methods

For this study, 30 frequently asked questions (FAQs) about asthma were collected from the Global Initiative for Asthma website (ginasthma.org). The chosen questions were organized into a list of six categories, with five questions in each category. The categories were as follows: general information about asthma, causes and trigger factors, symptoms, medical treatment, non-medical treatment, and a final section with questions about lifestyle and comorbidities. The ChatGPT application, version 3.5, was used for the evaluation as it is free of charge and broadly accessible to the general public [12].

On April 8th, 2024, the ChatGPT app was downloaded on two different devices by two different users in different locations for the sake of this study. This was done to avoid being influenced by previous user data. Each and every one of the questions was asked independently and separately in a new session through the "New Chat" feature. Again, this was done to avoid bias from previous conversations. The chatbot's initial response to each question was collected without response regeneration. On another device by a different user, the application was asked the same list of questions again in the same pattern, and both responses were collected to evaluate the consistency and reproducibility. An example of ChatGPT’s responses to one of the questions is displayed in Table 1. The full list of all questions and answers is provided in the Appendices section.

**Table 1:** An example of two responses by ChatGPT to the same question

Question asked	Response A	Response B
What are asthma controllers?	Asthma controllers are medications used on a regular basis to prevent asthma symptoms and attacks. They help reduce inflammation in the airways and make them less sensitive to triggers, thereby preventing or minimizing asthma symptoms. These medications are typically prescribed for individuals with persistent asthma and are different from quick-relief medications, which are used to treat sudden symptoms.	Asthma controllers are medications used to manage asthma symptoms and prevent asthma attacks. They work by reducing inflammation and swelling in the airways, making them less sensitive to triggers that can cause asthma symptoms. Controllers are typically taken regularly, even when asthma symptoms are not present, to keep asthma under control and prevent flare-ups. Examples include inhaled corticosteroids, long-acting beta-agonists, leukotriene modifiers, and mast cell stabilizers.

A set of five board-certified internal medicine physicians were asked to assess the two responses to each of the 30 questions. The evaluators were handed a spreadsheet containing instructions for the evaluation process as well as the 60 responses. The accuracy of each response was graded using a scale used in a previous study by Samaan et al. [13]: (1) comprehensive (accurate and comprehensive; nothing more a board-certified internal medicine physician would add to the response if asked this question by a patient); (2) correct but inadequate (all information is correct but incomplete, and a board-certified internal medicine physician would have more important information to add); (3) some correct and some incorrect; and (4) completely incorrect. Additionally, the acceptability of the responses was judged by asking the physicians a question: “Would this information be safe enough for you to share with your patient as the only answer to their question?” This aimed to assess whether the answers might miss vital details or could potentially pose a safety risk to patients.

The reliability of each response was described as “good” if at least four of the five physicians rated it as either “comprehensive” or “correct but inadequate.” Otherwise, it was considered “poor.” Reproducibility was assessed by comparing the final descriptive result of each of the two responses. If the two responses to the same question were either both “good” or both “poor” in terms of reliability, it was considered to be a consistent answer. Moreover, each response was considered acceptable if four or more physicians answered “yes” to the acceptability question [7]. Lastly, in order to quantify the qualitative ratings, a numerical scale was established by assigning values ranging from 1 to 4, corresponding to the qualitative grades of “completely incorrect” to “comprehensive,” respectively. The average rating of each response by the five raters was considered to resemble a consensus of evaluation. This transformation facilitated a more refined differentiation of the data, enabling a comprehensive analysis of the varying quality levels.

Statistical tests were chosen based on the normality of the data and employed to determine if there were any statistically significant differences in quantitative rating across the six categories of responses as well as assess the differences in reliability between the responses the first and second times each question was asked. A chi-square test was used to assess significant differences in the acceptability of the first and second sets of responses. Values of p<0.05 were considered statistically significant. SPSS Statistics version 27 (IBM Corp. Released 2020. IBM SPSS Statistics for Windows, Version 27.0. Armonk, NY: IBM Corp) was used for the analysis.

Finally, the readability of the sixty responses was evaluated utilizing the Flesch Reading Ease Scale (FRES), Flesch-Kincaid Grade Level (FKGL), and Simple Measure of Gobbledygook (SMOG) to find the mean and range of each measure. The acceptable readability levels were set at 80 or above for the FRES and below 7 for the FKGL and SMOG [14].

## Results

A total of 60 responses were collected, two responses per each of the 30 questions. Fifty-six (93.33%) of the responses were of good reliability. The average rating of the responses was 3.65 (95% CI 3.57 to 3.72) out of 4 total points. The overall acceptability of the responses was approximately 78% (n=47, 78.33%; 95% CI 67.60% to 89.06%). Table 2 shows the frequencies and proportions of the reliability and acceptability ratings for each category.

**Table 2:** The reliability and acceptability of ChatGPT's responses to asthma FAQs The data has been presented as frequencies (n) and proportions (%) FAQs: frequently asked questions a: responses in the category

	Reliability		Acceptability		
Category = n	Good n (%)	Poor n (%)	Yes n (%)		No n (%)
General information about asthma, n^a ^= 10	9 (90%)	1 (10%)	7 (70%)	3 (30%)	
Causes and trigger factors, n^a ^= 10	8 (80%)	2 (2%)	9 (90%)	1 (10%)	
Symptoms of asthma, n^a ^= 10	10 (100%)	0 (0%)	7 (70%)	3 (30%)	
Non-medical treatment, n^a ^= 10	10 (100%)	0 (0%)	8 (80%)	2 (20%)	
Medical treatment, n^a ^= 10	10 (100%)	0 (0%)	9 (90%)	1 (10%)	
Lifestyle and comorbidities, n^a ^= 10	9 (90%)	1 (10%)	7 (70%)	3 (30%)	
Total , n^a ^= 60	56 (93.33%)	4 (6.67%)	47 (78.33)	13 (21.67%)	

Only two (6.67%) of the 30 questions had inconsistent answers from the chatbot. There was no statistically significant difference in the reliability (t=1.673; p=0.275) of the first set of responses (3.687; 95% CI 3.593 to 3.780) and the second set of responses (3.607; 95% CI 3.491 to 3.722). There was no statistically significant difference in the acceptability (X2=0.545; p=0.460) of the first set of responses (n=25, 83.33%; 95% CI 69.18% to 97.49%) and the second set of responses (n=22, 73.33%; 95% CI 56.54% to 90.13%).

The readability of the 60 responses was determined to be, on average, 33.50 ±14.37 (difficult) on the FRES, 12.79±2.89 on the FKGL, and 13.47±2.38 on the SMOG. None of the responses had an acceptable readability score. The correlation coefficients between the reliability ratings and the FRES, FKGL, and SMOG readability scores of each response were 0.099, -0.092, and -0.136, respectively.

There was a significant difference (F(5, 5)=4.067, p=0.03) in the reliability of responses across the six different categories, with the responses to medical treatment questions being the highest rated (mean of 3.9±0.19). There was a statistically significant difference in the FRES (F(5, 5)=3.994, p=0.004) and the FKGL (F(5, 5)=4.465, p=0.002) of the responses among the six different categories. Table 3 shows the means of the reliability and readability ratings and their associations with question categories.

**Table 3:** The reliability and readability in association with question categories The data has been presented as means and standard deviations * Significant at p<0.05 level

	Category	General information about asthma	Causes and trigger factors	Symptoms of asthma	Non-medical treatment	Medical treatment	Lifestyle and comorbidities	Total average	P-value
Reliability	Average rating (range: 1-4)	3.52	3.58	3.7	3.72	3.9	3.46	3.65	0.03*
	Standard deviation of rating	0.316	0.209	0.271	0.215	0.194	0.268	0.281	
Readability	Flesch Reading Ease scale	34.37	40.168	38.865	40.269	26.463	20.874	33.502	0.004*
	Flesch-Kincaid Grade Level	12.798	11.221	12.823	10.616	14.142	15.113	12.786	0.002*
	Simple Measure of Gobbledygook	13.582	12.022	13.297	12.007	14.205	15.707	13.47	0.001*

## Discussion

Our study aimed to evaluate the reliability and acceptability of ChatGPT’s responses to 30 different questions across different categories, as well as assess the readability of the two sets of answers given to each question. Our assessment results show that 93.33% (n=56) of the responses generated by ChatGPT have been deemed “good” (“comprehensive” or “correct but inadequate"), with an average rating of 3.65 out of 4 points total. This suggests that the output of ChatGPT consistently provided reliable and comprehensive information, according to the evaluation done by the five assigned physicians. The overall acceptability of the responses was 78% (n=47), meaning that a significant portion of the responses were deemed acceptable information to convey to patients by the evaluators.

The chatbot exhibited high consistency in its responses. When presented with the same questions, it delivered answers that conveyed largely similar information. While a minority of responses with significantly more detail received higher ratings, most responses paraphrased each other, leading to low inconsistency rates. Statistical analysis (t-tests and chi-square) revealed no significant difference in the ratings between the first and second responses to each question. This suggests both responses achieved similar levels of reliability and acceptability, with only a few instances of inconsistency.

The highest-rated answers were to questions about medical treatment. This may be because, in addition to the accuracy of the information, the chatbot considered the concerns of the patient when answering. For example, after ChatGPT enumerated the side effects of inhaled steroids, it followed up with: “The benefits of controlling asthma symptoms generally outweigh the risks of these side effects when used appropriately under medical supervision.” This reassures the patients and encourages them to comply with the medication, as well as emphasizing the importance of medical supervision. Responses received lower ratings when they did not encourage shared decision-making with a medical professional.

In addition to lower reliability, responses that did not encourage patients to seek medical advice were considered to have lower acceptability as well. Moreover, responses to general questions about asthma received low acceptability ratings. This is because the evaluators believed that the responses, despite being accurate, were not adequate to be the only answers to such broad questions. It is rational to assume that a newly diagnosed patient, or a family member, would expect a longer, more detailed answer when they are first getting to know the disease. For example, the chatbot’s responses to questions such as “Can a person die from asthma?” were found unacceptable as they required further details and more support for the patient. Yet, as most responses were acceptable, we found this AI tool to be beneficial as a resource for patients to learn and acquire knowledge about the disease. We expect that this knowledge can contribute to a better health outcome.

Despite the overall good reliability and acceptability of ChatGPT’s responses, the readability scoring of those responses has been determined to be difficult on the FRES, FKGL test, and SMOG formula. None of the responses had an acceptable readability score due to ChatGPT using complex language that requires a higher level of knowledge and comprehension. Furthermore, a weak association was found between the reliability scores and the FRES, FKGL, and SMOG readability scores; responses rated higher on the reliability score had a slight tendency to score higher on the FRES and lower on the FKGL and SMOG tests.

There was, however, a significant difference in the reliability of responses across the six different categories, with the responses to medical treatment questions being the highest rated with an average of 3.9 out of 4 total points. This alone displays ChatGPT’s strength in providing reliable information in valuable areas and highlights its potential use in patient education. There was also a significant difference in the FRES and the FKGL scores among the responses to the different categories, with responses to lifestyle and comorbidity questions scoring the highest grade on the FKGL test, deeming it the most difficult answer to comprehend. On the other hand, responses to non-medical treatment questions scored the lowest grade on the FKGL test, possibly due to the chatbot using simpler and easier-to-understand concepts compared to other categories, yet still above the recommended level [14].

Our study results demonstrated that most of the answers were rated as “good” on reliability, which is similar to findings in a study conducted to evaluate ChatGPT’s responses to questions related to chronic diseases (diabetes, heart failure, chronic kidney disease, rheumatoid arthritis, and systemic lupus erythematosus) in Spanish with 83.3%, 50%, 66.6%, 83.3%, and 75%, respectively. The lower scoring of the study can possibly be attributed to the language used; given that ChatGPT has been trained in English, the chatbot's performance when asked in English is better compared to other languages [11].

Comparing ChatGPT’s responses’ readability to the results of a study done to evaluate asthma websites (Table 4), ChatGPT has scored an average reading ease score of 33.5 and a reading grade level score of 12.79, which significantly varies from the scores obtained by evaluating asthma websites with an average reading ease score of 53.57 and a reading grade level of 9.49 [16]. This demonstrates that the information provided by ChatGPT is of a higher reading level than traditional online resources and is substantially more difficult to read. However, ChatGPT’s responses and the information obtained from asthma websites are of higher reading difficulty levels than the recommended level determined by the United States Department of Health and Human Services [14].

**Table 4:** Readability of ChatGPT responses compared to previous studies assessing the readability of asthma education websites FKGL: Flesch-Kincaid Grade Level, SD: standard deviation

Study	Number of websites	FKGL	
		Mean (SD)	Range (min-max)
Reddy, et al, (2023) [15]	15	-	8.7-13.2
Banasiak, et al. (2017) [16]	22	9.5 (-)	2.9 -15.4
Banasiak, et al. (2013) [17]	6	9.7 (1.0)	8.0 - 10.3
Oermann, et al. (2003) [18]	70	9.2 (-)	2.7 -12.0
Croft, et al. (2002) [19]	90	10.3 (1.7)	-
ChatGPT	60 responses	12.8 (2.9)	7.3-18.6

We highlighted several strengths in this study. First, this is the first study conducted to evaluate ChatGPT's information regarding asthma. Second, a comprehensive evaluation of the responses was achieved by obtaining assessments from five different raters with a strong level of consensus between them. Third, comparisons of readability results were made based on previous studies done on websites for patient education on asthma; however, the reliability of those websites was not always assessed. Further studies can be made to directly compare the reliability and acceptability of ChatGPT to traditional website searches.

There are some limitations to our study that should be illuminated. First, this study was conducted on ChatGPT 3.5, which is freely available to the public and relies on information from a database of texts up to 2021. However, a newer version of ChatGPT (GPT-4) has been made available recently with a subscription model; this version is more advanced and has information up to April 2023. Therefore, the responses provided by ChatGPT 3.5 may be outdated in comparison. Further research should be done using ChatGPT 4 and its newer versions to evaluate the reliability and readability of its information [20].

Another limitation of our study was that the review of ChatGPT's responses was done by raters at an attending physician's level. Although this limitation was mended by asking rudimentary questions taken from the FAQ page of the GENA website, patients' age and level of education should be considered while evaluating these responses. In addition, the raters in this study were not blinded and had full knowledge that the responses were provided by ChatGPT, which could cause a bias in their assessment of the responses.

Lastly, it should be noted that only the first-generated responses were evaluated in this study. This method does not take into account the possibility of people asking the chatbot to simplify answers or ask for further clarification in which information could be corrected or supplemented in the ensuing answers. This aspect should be considered when conducting future research on the topic.

## Conclusions

Our study indicates that ChatGPT provides reliable responses to questions from asthma patients, particularly when providing information regarding medical treatments. Yet, challenges exist regarding the readability of these questions, limiting their accessibility to a certain set of users with higher education levels and health literacy. Despite this, ChatGPT remains a promising tool for patient education in conjunction with physicians’ advice. Addressing readability issues and further exploring its capabilities are essential for maximizing its effectiveness as a valuable resource in healthcare communication for asthma patients.

## Appendices

**Table 5:** A list of all questions and responses by ChatGPT to asthma FAQs FAQs: frequently asked questions

Question	Response A	Response B
What is asthma?	Asthma is a chronic respiratory condition characterized by inflammation and narrowing of the airways, leading to difficulty breathing, wheezing, coughing, and tightness in the chest. It can be triggered by various factors such as allergens, exercise, cold air, and respiratory infections. Asthma symptoms can range from mild to severe and can be managed with medications and lifestyle changes.	Asthma is a chronic respiratory condition characterized by inflammation and narrowing of the airways, which leads to difficulty breathing, coughing, wheezing, and tightness in the chest. It can be triggered by various factors such as allergies, respiratory infections, exercise, cold air, or pollutants. Asthma attacks can range from mild to severe and may require medical intervention to manage symptoms and prevent complications.
Is asthma a chronic disease?	Yes, asthma is considered a chronic disease. It is a condition that affects the airways in the lungs and can cause recurring episodes of wheezing, breathlessness, chest tightness, and coughing. These symptoms can vary in severity and frequency over time.	Yes, asthma is considered a chronic respiratory condition characterized by inflammation and narrowing of the airways, leading to symptoms like wheezing, shortness of breath, chest tightness, and coughing. It's a long-term condition that requires ongoing management and treatment to control symptoms and prevent flare-ups.
What causes asthma?	Asthma is caused by a combination of genetic and environmental factors. Common triggers include allergens (like pollen, dust mites, or pet dander), respiratory infections, irritants (such as smoke or pollution), exercise, cold air, and certain medications.	Asthma is primarily caused by a combination of genetic and environmental factors. Common triggers include allergens like pollen, dust mites, pet dander, respiratory infections, air pollution, tobacco smoke, and certain medications. Additionally, factors like genetics, respiratory infections during childhood, and early exposure to allergens can increase the risk of developing asthma.
At what age does asthma start?	Asthma can develop at any age, but it often starts in childhood. It can also develop for the first time in adulthood. However, symptoms may vary depending on the age of onset and other factors.	Asthma can start at any age, but it's most commonly diagnosed in childhood. However, some people develop asthma for the first time as adults.
Is there a risk that my asthma will get worse with age?	Asthma can vary in severity over time, and while some people experience improvement as they age, others may find their symptoms worsen. It's essential to work closely with your healthcare provider to manage your asthma effectively and monitor any changes in symptoms. Regular check-ups and adhering to your treatment plan can help minimize the risk of worsening symptoms.	Asthma symptoms can vary over time, and while some people may find their asthma improves or remains stable as they age, others might experience worsening symptoms. Aging can lead to changes in lung function and overall health, which may impact asthma severity. It's essential to work closely with your healthcare provider to manage your asthma effectively as you age and adjust treatment as needed. Regular check-ups and monitoring can help identify any changes in your condition early on.
What are the main asthma triggers?	Asthma triggers can vary from person to person, but some common ones include: 1. Allergens such as pollen, dust mites, pet dander, and mold. 2. Irritants like smoke, air pollution, strong odors, and perfumes. 3. Respiratory infections, such as colds and flu. 4. Physical activity or exercise-induced asthma. 5. Weather changes, particularly cold air. 6. Strong emotions or stress. 7. Certain medications or food additives. 8. Gastroesophageal reflux disease (GERD). 9. Hormonal changes, such as those during menstruation or pregnancy. Identifying and managing triggers is crucial in asthma management.	Asthma triggers can vary widely among individuals, but some of the main ones include: 1. Allergens such as pollen, mold, pet dander, and dust mites. 2. Respiratory infections, like the common cold or the flu. 3. Physical activity, especially in cold and dry air. 4. Cold air or sudden changes in weather. 5. Tobacco smoke, including secondhand smoke. 6. Air pollution and outdoor pollutants like ozone, as well as indoor pollutants such as certain chemicals. 7. Strong emotions and stress, which can lead to hyperventilation and tightening of the airways. 8. Certain medications, including beta-blockers, aspirin, and nonsteroidal anti-inflammatory drugs (NSAIDs). 9. Food additives, in rare cases, such as sulfites used in dried fruit, wine, and processed foods. 10. Gastroesophageal reflux disease (GERD), a condition where stomach acid flows back into the throat, which can irritate the airways. Identifying and avoiding personal asthma triggers is a key part of managing the condition effectively.
Can weather changes trigger asthma?	Yes, weather changes can trigger asthma symptoms in some people. Factors such as cold air, hot and humid conditions, changes in temperature, high pollen levels, and air pollution can all contribute to asthma flare-ups. Cold, dry air can irritate the airways and trigger bronchospasms, while hot and humid conditions can increase the presence of allergens like mold and pollen in the air. It's important for individuals with asthma to be aware of weather changes and take necessary precautions to manage their condition, such as using inhalers as prescribed and avoiding triggers when possible.	Yes, weather changes can indeed trigger asthma symptoms in some people. Rapid changes in temperature, cold air, hot and humid conditions, and windy or stormy weather can all affect air quality and irritate the airways, leading to asthma flare-ups. It's important for those with asthma to be aware of the weather conditions that might affect them and to manage their condition accordingly, often with the advice of a healthcare provider.
Can infections trigger asthma?	Yes, infections such as colds or respiratory viruses can trigger asthma symptoms or exacerbate existing asthma. This is because the inflammation and irritation caused by infections can narrow the airways, making it harder to breathe for people with asthma.	Yes, respiratory infections are known triggers for asthma. Viral infections, such as the common cold, flu, and respiratory syncytial virus (RSV), are among the most common triggers, especially in children. These infections can cause inflammation and narrowing of the airways, leading to asthma symptoms like wheezing, coughing, shortness of breath, and chest tightness. Managing asthma often involves avoiding known triggers, including infections, and following a treatment plan prescribed by a healthcare provider.
Can medications trigger asthma?	Yes, certain medications can trigger asthma symptoms in some people. These medications are known as "asthma triggers" and can include aspirin, nonsteroidal anti-inflammatory drugs (NSAIDs) like ibuprofen, beta-blockers, and some medications used to treat high blood pressure or heart conditions. It's important for individuals with asthma to discuss potential triggers with their healthcare provider.	Yes, certain medications can trigger asthma or worsen symptoms for some people. Common examples include: 1. Beta-blockers: Used for heart disease, high blood pressure, and migraines. They can cause airway constriction that can lead to asthma symptoms. 2. Aspirin and Non-Steroidal Anti-Inflammatory Drugs (NSAIDs): Some individuals, particularly adults with asthma, may experience worsened symptoms when taking aspirin or NSAIDs like ibuprofen. 3. Angiotensin-Converting Enzyme (ACE) Inhibitors: Used for high blood pressure and heart failure; they can cause coughing, which may aggravate asthma in some people. It's important for individuals with asthma to inform their healthcare providers about their condition so they can consider it when prescribing medications.
What chemicals, irritants, or other substances trigger asthma?	Asthma triggers can vary from person to person, but common ones include: 1. Allergens such as pollen, dust mites, pet dander, mold spores. 2. Irritants like tobacco smoke, air pollution, strong odors, and chemicals. 3. Respiratory infections such as colds and flu. 4. Physical activity, especially in cold or dry air. 5. Weather changes like cold air or sudden temperature shifts. 6. Certain medications like aspirin or nonsteroidal anti-inflammatory drugs (NSAIDs). 7. Emotional stress or strong emotions. 8. Gastroesophageal reflux disease (GERD), which can trigger asthma symptoms in some individuals.	Asthma triggers can vary significantly among individuals, but there are several common irritants and substances known to potentially exacerbate asthma symptoms: 1. Allergens: - Pollen from trees, grasses, and weeds - Dust mites - Pet dander from animals like cats and dogs - Mold spores - Cockroach droppings 2. Respiratory Infections: Viruses like the common cold or the flu. 3. Air Pollutants and Irritants: - Tobacco smoke - Air pollution (e.g., ozone and particulate matter) - Workplace irritants (e.g., chemical fumes, gases, or dust) 4. Physical Activity: Exercise, especially in cold and dry air. 5. Weather: Changes in weather, cold air, humidity, and stormy weather. 6. Strong Emotions: Stress and strong emotions can lead to hyperventilation and tightening of the airways. 7. Medications: - Aspirin and nonsteroidal anti-inflammatory drugs (NSAIDs) - Beta-blockers (used for heart disease, high blood pressure, and migraines) 8. Foods and Food Additives: - Sulfites and preservatives added to some types of food and beverages, including wine, dried fruit, and processed potatoes. - Some people may have allergies to certain foods like peanuts, shellfish, eggs, or dairy that can trigger symptoms. 9. Gastroesophageal Reflux Disease (GERD): Stomach acids backing up into the throat can trigger or worsen asthma symptoms. 10. Fragrances and Volatile Organic Compounds (VOCs): Household cleaners, scented products, and paints. Understanding and avoiding personal triggers is a key strategy in managing asthma effectively. This often involves monitoring symptoms in relation to exposure to potential triggers and making lifestyle or environmental changes to reduce exposure.
What does asthma feel like?	Asthma can feel different for everyone, but common symptoms include shortness of breath, wheezing, chest tightness, and coughing. During an asthma attack, it can feel like there's a heavy weight on your chest, making it difficult to breathe deeply. It can also cause anxiety and panic due to the sensation of struggling to get enough air.	Asthma can feel like tightness or pressure in the chest, shortness of breath, wheezing (a whistling sound when breathing), and coughing, especially at night or early in the morning. It can vary from person to person and from one asthma episode to another. Some people may experience mild symptoms, while others may have more severe breathing difficulties.
What happens during an asthma attack?	During an asthma attack, the airways become inflamed and narrowed, causing difficulty in breathing. This inflammation leads to increased mucus production and muscle constriction around the airways, making it harder for air to pass through. Symptoms may include wheezing, coughing, shortness of breath, and chest tightness. Asthma attacks can be triggered by various factors such as allergens, exercise, respiratory infections, or exposure to irritants like smoke or pollution.	During an asthma attack, the airways in the lungs become inflamed and swollen, making it difficult to breathe. This inflammation leads to the production of excess mucus, further narrowing the air passages. Symptoms can include wheezing, coughing, shortness of breath, and chest tightness. In severe cases, it can be life-threatening and require immediate medical attention.
Can a person die from asthma?	Yes, in severe cases, asthma attacks can be fatal if not properly managed or treated promptly. While most asthma-related deaths are preventable with proper medication and management, it's essential for individuals with asthma to have an asthma action plan in place and to seek medical attention if experiencing severe symptoms.	Yes, in severe cases, asthma can be life-threatening if not properly managed or during an asthma attack, especially if medical attention is not sought promptly.
What are the signs of a severe and dangerous asthma attack?	Signs of a severe and dangerous asthma attack include: 1. Wheezing: Continuous or worsening wheezing despite medication. 2. Shortness of breath: Difficulty breathing, especially when at rest or during minimal activity. 3. Coughing: Persistent coughing that doesn't improve with usual medications. 4. Chest tightness: Feeling of pressure or constriction in the chest. 5. Difficulty speaking: Inability to speak in full sentences due to shortness of breath. 6. Blue lips or fingernails: A sign of oxygen deprivation. 7. Increased heart rate: Rapid or irregular heartbeat. 8. Use of accessory muscles: Visible use of neck or chest muscles to breathe. 9. Extreme anxiety: Feeling panicked or fearful due to difficulty breathing. 10. No improvement with rescue inhaler: If a rescue inhaler doesn't provide relief or symptoms worsen after use, it's a sign of a severe attack. If experiencing any of these symptoms, seeking immediate medical attention is crucial.	Signs of a severe and dangerous asthma attack include extreme difficulty breathing, rapid breathing, wheezing that persists despite medication, inability to speak more than short phrases due to breathlessness, a bluish tint to the lips or fingernails (cyanosis), and a feeling of tightness in the chest that doesn't improve with medication. If you notice these symptoms, seek immediate medical attention or call emergency services.
Can asthma medication help prevent asthma symptoms?	Yes, asthma medication can help prevent asthma symptoms by reducing inflammation and narrowing of the airways, which are the underlying causes of asthma symptoms like wheezing, coughing, and shortness of breath. These medications can include inhaled corticosteroids, long-acting beta agonists, leukotriene modifiers, and others, depending on the severity and type of asthma. It's important for individuals with asthma to work with their healthcare provider to develop a personalized treatment plan to effectively manage their symptoms and prevent asthma attacks.	Yes, asthma medications are designed to prevent and relieve asthma symptoms. There are two main types: 1. Controller medications: These are taken daily to prevent asthma symptoms and attacks by reducing inflammation and mucus production in the airways. 2. Reliever medications: These are used as needed to quickly relieve symptoms like wheezing and shortness of breath during an asthma attack by relaxing the muscles around the airways. It's crucial for asthma patients to work with their healthcare provider to develop an effective treatment plan tailored to their specific needs.
I have asthma, how can I avoid common triggers?	To avoid common asthma triggers, consider these tips: 1. Allergens: Keep your living space clean, vacuum regularly, use dust mite covers on bedding, and avoid exposure to pollen by keeping windows closed during high pollen seasons. 2. Smoke: Avoid exposure to cigarette smoke, and stay away from areas where people are smoking. 3. Air Pollution: Check air quality forecasts and avoid outdoor activities during high pollution days. Consider using air purifiers indoors. 4. Cold Air: Cover your nose and mouth with a scarf in cold weather and consider wearing a mask if needed. 5. Exercise: Warm up before exercising and try activities less likely to trigger asthma, such as swimming or walking. 6. Respiratory Infections: Wash hands frequently and avoid close contact with sick individuals to reduce the risk of respiratory infections. 7. Stress: Practice stress-reducing techniques such as deep breathing exercises or yoga to manage asthma symptoms triggered by stress. 8. Pets: Keep pets out of the bedroom, bathe pets regularly, and vacuum frequently to reduce exposure to pet dander. 9. Strong Odors: Avoid strong perfumes, scented candles, and other strong odors that may trigger asthma symptoms. 10. Weather Changes: Pay attention to weather changes and take necessary precautions, such as using a humidifier in dry conditions. Remember to work closely with your healthcare provider to develop a personalized asthma action plan tailored to your specific triggers and needs.	Managing asthma involves being proactive about avoiding triggers. Here are some common triggers and ways to avoid them: 1. Allergens: These can include pollen, dust mites, mold, pet dander, and cockroach droppings. - Solution: Use allergen-proof mattress and pillow covers, wash bedding in hot water weekly, use a high-efficiency particulate air (HEPA) filter in your vacuum cleaner and air purifier, and keep humidity levels low to prevent mold growth. For pet dander, consider not allowing pets in bedrooms or on upholstered furniture. 2. Tobacco Smoke: Smoke is a powerful asthma trigger. - Solution: Avoid smoking and secondhand smoke. If you smoke, seek help to quit. Avoid areas where smoking is allowed. 3. Air Pollution: This includes outdoor pollution like smog, ozone, and car exhaust, as well as indoor sources like fragrances or cleaning products. - Solution: Check air quality forecasts and limit outdoor activities when air quality is poor. Use unscented cleaning products and avoid aerosol sprays. 4. Cold Air: Cold air can tighten airways. - Solution: Wear a scarf or a mask over your nose and mouth if you have to be outside in cold weather. 5. Exercise: While exercise is important for overall health, it can sometimes trigger asthma symptoms. - Solution: Warm up before starting exercise and cool down afterward. If cold air triggers your asthma, try to exercise indoors during cold weather. There are also medications that can be used pre-exercise to prevent symptoms. 6. Stress and Emotions: Stress and strong emotions can sometimes lead to asthma flare-ups. - Solution: Practice stress-reduction techniques such as deep breathing, yoga, meditation, or progressive muscle relaxation. 7. Sulfites in Food: Some people with asthma are sensitive to sulfites, preservatives added to some foods and drinks. - Solution: Avoid foods and drinks that contain sulfites, such as dried fruits, wine, and processed potatoes. 8. Infections: Respiratory infections can trigger asthma. - Solution: Wash your hands frequently, avoid close contact with sick people, and get vaccinated against the flu and pneumonia. Regularly cleaning your home to remove dust, avoiding the use of heavy perfumes or air fresheners, and maintaining a healthy diet and lifestyle can also help control asthma symptoms. It's crucial to work with your healthcare provider to create a personalized asthma action plan tailored to your specific triggers and needs.
I have asthma and I am allergic to pets, what can I do?	Living with asthma and pet allergies can be challenging, but there are steps you can take to manage your condition: 1. Avoid Exposure: Try to minimize your contact with pets, especially those you are allergic to. This might involve avoiding homes with pets or limiting your time spent around animals. 2. Create Pet-Free Zones: Designate certain areas of your home as pet-free zones, such as your bedroom, to reduce your exposure to allergens while you sleep. 3. Use Air Purifiers: Consider using high-efficiency particulate air (HEPA) filters in your home to help remove pet dander and other allergens from the air. 4. Keep Pets Outdoors: If you have pets, keep them outdoors as much as possible to reduce the amount of pet dander in your living space. 5. Regular Cleaning: Vacuum your home frequently with a vacuum cleaner equipped with a HEPA filter. Wash bedding, curtains, and other fabrics regularly to remove pet dander. 6. Medication: Talk to your doctor about appropriate medications to manage your asthma and allergies, such as antihistamines or corticosteroids. They may also prescribe a rescue inhaler for asthma attacks. 7. Allergy Shots: Consider immunotherapy (allergy shots) to help reduce your sensitivity to pet allergens over time. This involves receiving regular injections of small amounts of allergens to desensitize your immune system. 8. Consult an Allergist: If your symptoms are severe or difficult to manage, consider consulting an allergist for personalized advice and treatment options. By taking these steps, you can help minimize your exposure to pet allergens and better manage your asthma and allergies.	Managing asthma and pet allergies can be challenging, but there are steps you can take to minimize symptoms and exposure. Here are some strategies: 1. Allergy Testing: Confirm your specific allergies with an allergist. This can help tailor your avoidance strategies and treatments. 2. Pet-Free Zones: Designate certain areas of your home, like your bedroom, as pet-free zones to reduce allergen exposure. 3. HEPA Filters: Use HEPA (High-Efficiency Particulate Air) filters in your vacuum cleaner and air purifiers to capture pet dander and other allergens. 4. Frequent Cleaning: Clean your home regularly, including floors, furniture, and other surfaces where pet dander can accumulate. Wash bedding and upholstery often. 5. Pet Grooming: Regularly bathe and groom your pet to reduce the amount of dander. Doing this outside your home or using professional grooming services can further minimize your exposure. 6. Medications: Discuss with your doctor or allergist about medications that can help control your allergic reactions and asthma symptoms. This might include antihistamines, nasal sprays, inhalers, or even allergy shots (immunotherapy). 7. Air Circulation: Improve the ventilation in your home. Opening windows when possible and using exhaust fans can help reduce the concentration of indoor allergens. 8. Hand Washing: After handling your pet, wash your hands thoroughly. This simple step can reduce the spread of allergens to your eyes and nose. 9. Hypoallergenic Pets: If you don’t yet have a pet but are considering one, look into breeds that are less likely to trigger allergies. However, no pet is 100% hypoallergenic, so spend time with the animal before making a decision. 10. Consult an Allergist: An allergist can provide personalized advice and treatment options, which may include medications, lifestyle changes, or allergy shots. 11. Educate Yourself and Others: Learn as much as you can about managing asthma and allergies, and educate those around you, especially family members, about your condition and how they can help reduce your exposure to allergens. Remember, individual sensitivity to allergens can vary greatly, so it's essential to find the right balance of exposure and management that works for you.
I have asthma and I am allergic to dust mites, what can I do?	Managing asthma when allergic to dust mites involves several steps: 1. Reduce Dust Mites: Use allergen-proof covers on mattresses and pillows, wash bedding weekly in hot water, keep humidity levels low, and regularly vacuum carpets and upholstery. 2. Medication: Take prescribed asthma medications, including inhalers and allergy medications, as directed by your doctor. 3. Avoid Triggers: Minimize exposure to other asthma triggers such as tobacco smoke, pet dander, and air pollution. 4. Regular Check-ups: Schedule regular check-ups with your healthcare provider to monitor your asthma and allergy symptoms. 5. Emergency Plan: Have an asthma action plan in place, including knowing when to seek emergency medical attention if symptoms worsen. 6. Healthy Lifestyle: Maintain a healthy lifestyle with regular exercise, a balanced diet, and stress management techniques to support overall respiratory health.	Managing asthma when you're allergic to dust mites involves several steps: 1. Reduce Exposure: Use allergen-proof mattress and pillow covers, wash bedding weekly in hot water, and vacuum carpets and upholstery regularly. 2. Maintain Cleanliness: Keep your home clean and dust-free, especially in areas where dust mites thrive, like carpets, curtains, and upholstery. 3. Medication: Take prescribed asthma medications as directed by your doctor, including rescue inhalers and controller medications to manage symptoms and prevent flare-ups. 4. Allergy Medications: Consider allergy medications such as antihistamines or nasal corticosteroids to manage allergic reactions to dust mites. 5. Consult with an Allergist: They can provide allergy testing and advise on specific avoidance measures and treatment options tailored to your needs. 6. Healthy Lifestyle: Maintain a healthy lifestyle with regular exercise, balanced nutrition, and avoiding smoking or exposure to secondhand smoke, as these factors can worsen asthma symptoms. 7. Stay Informed: Keep up-to-date with asthma management techniques and new treatments by consulting with healthcare providers and reliable sources of information.
Can I reduce my asthma if I get an air purifier?	An air purifier can help reduce asthma triggers like dust, pollen, and pet dander, potentially improving your symptoms. However, it's not a guaranteed solution. It's essential to also manage other factors like medication, allergen control, and regular medical check-ups for effective asthma management.	Yes, an air purifier can help reduce asthma symptoms by removing allergens and pollutants from the air, such as dust mites, pet dander, pollen, and smoke. These are common triggers for asthma attacks. However, it's important to choose an air purifier with a HEPA filter for it to be effective in capturing small particles. Additionally, maintaining a clean environment and following your asthma management plan as prescribed by your healthcare provider are crucial steps in managing your asthma effectively.
I have asthma, what can I do to improve my home environment in general?	Improving your home environment can help manage asthma symptoms. Here are some tips: 1. Keep it Clean: Regularly dust, vacuum, and mop to reduce dust mites, pet dander, and other allergens. 2. Control Humidity: Use a dehumidifier to keep humidity levels between 30-50% to prevent mold growth. 3. Reduce Allergens: Use allergen-proof covers on pillows and mattresses. Wash bedding regularly in hot water. 4. Air Purifiers: Consider using HEPA air purifiers to remove airborne allergens and pollutants. 5. Ventilation: Ensure good ventilation in your home to reduce indoor air pollutants. Use exhaust fans in bathrooms and kitchens. 6. Avoid Smoke: Keep your home smoke-free, including tobacco smoke and wood-burning stoves. 7. Minimize Chemicals: Use fragrance-free and hypoallergenic cleaning products to reduce exposure to irritants. 8. Control Pests: Keep food stored in airtight containers and fix any leaks to prevent pests like cockroaches and rodents. 9. Regular Maintenance: Maintain your HVAC system and change air filters regularly to improve air quality. 10. Keep Pets Clean: Bathe and groom pets regularly to reduce dander and fur in your home. 11. Monitor Indoor Air Quality: Consider getting an indoor air quality monitor to track pollutants and allergens in your home. 12. Consult a Professional: If you're unsure about the best steps to take, consider consulting with an allergist or environmental specialist for personalized advice.	Improving your home environment can help manage asthma symptoms. Here are some tips: 1. Keep it clean: Regularly dust, vacuum, and mop to reduce dust mites and other allergens. 2. Control humidity: Use a dehumidifier to keep humidity levels between 30-50% to prevent mold growth. 3. Ventilation: Ensure good airflow by opening windows and using exhaust fans in bathrooms and kitchens. 4. Remove carpets: Consider replacing carpets with hard flooring to reduce dust accumulation. 5. Avoid smoking indoors: Secondhand smoke can trigger asthma symptoms. 6. Control pests: Use traps or professional pest control to minimize exposure to cockroaches and other pests. 7. Wash bedding: Wash bedding weekly in hot water to kill dust mites. 8. Filter air: Use high-efficiency particulate air (HEPA) filters in your HVAC system to trap allergens. Consult with your doctor for personalized advice on managing asthma in your home environment.
What are asthma controllers?	Asthma controllers are medications used on a regular basis to prevent asthma symptoms and attacks. They help reduce inflammation in the airways and make them less sensitive to triggers, thereby preventing or minimizing asthma symptoms. These medications are typically prescribed for individuals with persistent asthma and are different from quick-relief medications, which are used to treat sudden symptoms.	Asthma controllers are medications used to manage asthma symptoms and prevent asthma attacks. They work by reducing inflammation and swelling in the airways, making them less sensitive to triggers that can cause asthma symptoms. Controllers are typically taken regularly, even when asthma symptoms are not present, to keep asthma under control and prevent flare-ups. Examples include inhaled corticosteroids, long-acting beta-agonists, leukotriene modifiers, and mast cell stabilizers.
What are asthma relievers?	Asthma relievers, also known as rescue inhalers, are medications used to quickly relieve asthma symptoms like shortness of breath, wheezing, and chest tightness. They work by relaxing the muscles around the airways, making it easier to breathe. The most common type of reliever medication is a short-acting beta-agonist (SABA), such as albuterol. These inhalers provide rapid relief during asthma attacks or when symptoms worsen. It's important for people with asthma to carry their reliever inhaler with them at all times for immediate use when needed.	Asthma relievers, also known as rescue inhalers, are medications that provide quick relief from asthma symptoms like shortness of breath, wheezing, and chest tightness. They typically contain short-acting beta-agonists (SABAs) like albuterol or levalbuterol, which work by relaxing the muscles around the airways, making breathing easier. These inhalers are used as needed during asthma attacks or when symptoms flare up suddenly. It's important to follow your doctor's instructions for proper usage and to have your reliever inhaler with you at all times if you have asthma.
I have asthma, what are inhaled glucocorticosteroids?	Inhaled glucocorticosteroids are medications used to treat asthma by reducing inflammation in the airways. They work by decreasing swelling and mucus production in the air passages, making it easier to breathe. These medications are typically delivered through inhalers and are considered a mainstay in asthma management for long-term control of symptoms and prevention of asthma attacks.	Inhaled glucocorticosteroids are medications used to manage asthma by reducing inflammation in the airways. They work by inhibiting the production of inflammatory chemicals, thereby decreasing swelling and mucus production in the airways. This helps to prevent asthma symptoms such as wheezing, coughing, and shortness of breath. Some common inhaled glucocorticosteroids include fluticasone, budesonide, and beclomethasone. They are typically used as a long-term controller medication in asthma management.
Do inhaled corticosteroids cause any long-term side effects for an asthmatic patient?	Long-term use of inhaled corticosteroids for asthma management can lead to potential side effects such as oral thrush, hoarseness, and, in rare cases, bone density loss or cataracts. However, the benefits of controlling asthma symptoms generally outweigh the risks of these side effects when used appropriately under medical supervision. Regular monitoring and proper use of spacer devices can help minimize these risks. It's crucial for patients to discuss any concerns with their healthcare provider.	Inhaled corticosteroids (ICS) are generally safe and effective for managing asthma when used as prescribed. However, long-term use at high doses can potentially lead to side effects such as oral thrush, hoarseness, and a slight increase in the risk of cataracts and osteoporosis. It's important for patients to use ICS as directed by their healthcare provider and to regularly monitor for any adverse effects.
Do I still need my inhaled corticosteroid for asthma if I feel OK?	It's important to continue taking your inhaled corticosteroid medication as prescribed, even if you're feeling fine. This medication helps to control inflammation in your airways and prevent asthma symptoms from worsening, even when you're not experiencing symptoms. Always follow your healthcare provider's advice regarding your asthma medication. If you have any concerns or questions about your treatment plan, it's best to discuss them with your healthcare provider.	Yes, it's essential to continue using your inhaled corticosteroid medication as prescribed by your doctor, even if you feel okay. This helps prevent asthma symptoms and keeps inflammation in check, reducing the risk of flare-ups or exacerbations in the future. Always consult your healthcare provider before making any changes to your medication regimen.
Are relaxing exercises good for my asthma?	Relaxing exercises, such as deep breathing and gentle yoga, can help manage asthma by reducing stress and improving lung function. However, it's important to consult with your healthcare provider to determine the most appropriate exercises for your specific condition and to ensure they are safe for you.	Yes, relaxing exercises like deep breathing, yoga, and tai chi can help manage asthma by reducing stress and improving breathing techniques. However, it's important to consult with your healthcare provider to ensure these exercises are safe and suitable for your individual condition.
How does asthma affect my choice of a professional career?	Asthma can influence your career choices depending on the nature of the job and your asthma severity. Careers involving exposure to triggers like dust, chemicals, or extreme temperatures might pose challenges. However, many careers are asthma-friendly, such as office-based jobs, teaching, writing, or remote work. It's essential to consider your asthma management plan and any accommodations needed when selecting a career path.	Asthma can influence your career choices depending on factors like triggers, environmental conditions, and physical demands. Some careers may expose you to substances that worsen asthma symptoms, like dust or chemicals, making them less suitable. Jobs with physical exertion or high-stress levels might also be challenging if they trigger asthma attacks. It's essential to consider these factors and choose a career with environments and conditions conducive to managing your asthma effectively. Additionally, having accommodations and understanding colleagues can make a significant difference in navigating your professional path with asthma. Consulting with healthcare professionals can provide insights tailored to your specific condition and career aspirations.
Is asthma a psychological (psychosomatic) disease?	Asthma is primarily a physical condition involving inflammation and narrowing of the airways, typically triggered by environmental factors like allergens or respiratory infections. However, psychological factors such as stress and anxiety can sometimes exacerbate symptoms or trigger asthma attacks. So while asthma is not solely a psychological disease, psychological factors can play a role in its management and impact on an individual's health.	Asthma is primarily a physical condition involving inflammation and narrowing of the airways, leading to difficulty in breathing. However, psychological factors like stress can exacerbate symptoms in some individuals, but it's not considered a purely psychological or psychosomatic disease.
Is emphysema the same as asthma?	No, emphysema and asthma are not the same. Asthma is a chronic inflammatory condition of the airways characterized by episodes of wheezing, breathlessness, chest tightness, and coughing. It often starts in childhood and can be triggered by various factors such as allergens, exercise, or respiratory infections. Emphysema, on the other hand, is a type of chronic obstructive pulmonary disease (COPD) characterized by damage to the air sacs in the lungs, leading to difficulty in breathing. It is commonly caused by long-term exposure to irritants such as cigarette smoke and is typically seen in older individuals. While both conditions affect breathing, they have different causes, mechanisms, and treatments.	No, emphysema and asthma are not the same. Emphysema is a chronic lung condition primarily caused by smoking or long-term exposure to air pollution, leading to damage to the air sacs in the lungs. Asthma, on the other hand, is a chronic inflammatory condition of the airways that can be triggered by various factors such as allergens, exercise, or respiratory infections. They have different causes, symptoms, and treatments.
Is asthma related to other chronic diseases, such as rheumatism and diabetes?	Asthma can be associated with other chronic diseases like rheumatoid arthritis and diabetes. While they may seem unrelated, they can share underlying immune system dysregulation or inflammatory processes. However, having one of these conditions doesn't necessarily mean you'll have the others, but it's essential to manage each condition properly as they can interact and affect one another.	While asthma, rheumatism (arthritis), and diabetes are all chronic conditions, they aren't directly related in terms of causation. However, there can be overlapping risk factors or shared underlying immune system dysregulation. For instance, inflammation plays a role in all three conditions, and certain factors like obesity can exacerbate both asthma and diabetes. Each condition requires its own management and treatment approach. It's essential to work closely with healthcare providers to address the specific needs of each condition.
